# Analysis of systemic serum vancomycin levels following intraarticular application in primary total joint arthroplasty

**DOI:** 10.1007/s00402-024-05688-6

**Published:** 2024-12-18

**Authors:** Stauss R, Savov P, Seeber GH, Brand S, Ettinger M, Beheshty JA

**Affiliations:** 1 School of Medicine and Health Sciences, Division of Orthopaedics at Campus Pius-Hospital, Carl von Ossietzky Universität Oldenburg, Georgstraße 12, 26121 Oldenburg, Germany; 2 Department of Orthopedics, University of Groningen, University Medical Center Groningen, Groningen, The Netherlands

**Keywords:** Total knee arthroplasty, Total hip arthroplasty, Total joint arthroplasty, Periprosthetic joint infection, Vancomycin powder

## Abstract

**Introduction:**

Periprosthetic joint infection (PJI) is a serious complication following primary total joint arthroplasty (TJA). PJI accounts for 15–25% of revision surgeries, therefore it is associated with PJI is associated with substantial patient morbidity and mortality as well as increased healthcare expenditures due to complex treatment strategies.

Recently, intraoperative local application of vancomycin powder is increasingly being used in primary total hip and knee arthroplasty (THA, TKA) as an additive strategy for PJI prevention. Whereas local vancomycin concentrations have already been investigated in prior studies, evidence on systemic vancomycin levels and potential adverse drug reactions (ADR) is limited. Purpose of this study was to investigate systemic vancomycin levels following intraarticular application in primary TJA.

**Materials and methods:**

This pilot study is a prospective analysis of patients undergoing primary THA and TKA between April and July 2023. One gram of vancomycin powder was applied to the prosthesis prior to wound closure. Serum vancomycin levels were measured at two standardised time points, 24 and 48 h postoperatively.

**Results:**

In total, 103 patients were included, and the patient collective was further stratified by surgical procedure into a THA subgroup (n = 52) and a TKA subgroup (n = 51). Mean serum vancomycin levels showed a significant group difference at both time points (24 h: p < 0.001; 48 h: p = 0.044) with higher serum vancomycin concentrations in the THA cohort. Mean serum vancomycin levels in THA patients were 1.25 μg/ml (range 0.00–7.00 μg/ml) after 24 h and 0.34 μg/ml (range 0.00–4.80 μg/ml) 48 h postoperatively. In TKA, no systemic vancomycin levels were detected. Vancomycin concentrations did not reach therapeutic levels in any patient. No ADR was detected in the whole study collective.

**Conclusion:**

Following intraarticular administration of vancomycin powder, no systemic vancomycin levels within the therapeutic range were detected, thus it may serve as a safe and cost-effective adjunct to strategies for prevention of PJI.

## Introduction

Periprosthetic joint infection (PJI) is one of the most devastating complications of primary total joint arthroplasty (TJA) as it may cause substantial loss of function, morbidity and mortality [1–3]. Being among the most common causes for revision, PJI does not only pose a surgical challenge, but is also associated with increased health care expenditures due to complex treatment strategies [4–7]. Despite all perioperative improvements, PJI rates remain at a constant level of 1–2% [8, 9]. Given the predicted increasing demand for total hip and total knee arthroplasties in the coming decades, a consecutive increase in periprosthetic infections is expected [9, 10]. To counteract this, there is a significant interest in additional prevention strategies to reduce the incidence of PJI.

The current literature shows a trend towards an increased use of local antibiotics in orthopedic surgery. In the field of spinal and trauma surgery, the use of intrawound vancomycin powder (VP) has demonstrated efficacy as it significantly reduced surgical site infection rates (SSI) [11–13]. In primary total joint arthroplasty, intraarticular application of vancomycin powder (VP) is increasingly being used as an additive strategy for PJI prevention. Local administration of antibiotics leads to high tissue concentrations at the operative site while potentially avoiding systemic side effects [14, 15]. First evidence is promising, as a significant decrease in PJI rates is reported for patients who additionally received local VP compared to a control group [16–19]. These results suggest that local VP application may be a safe and cost-effective strategy for PJI prevention. However, evidence on systemic vancomycin levels and potential adverse drug reactions (ADR) is limited.

The purpose of this pilot study was to investigate systemic vancomycin levels following intraarticular application of vancomycin powder in primary TJA. We hypothesized that topic application of vancomycin powder would not result in systemic vancomycin levels within the therapeutic range.

## Materials and methods

**Fig. 1:**
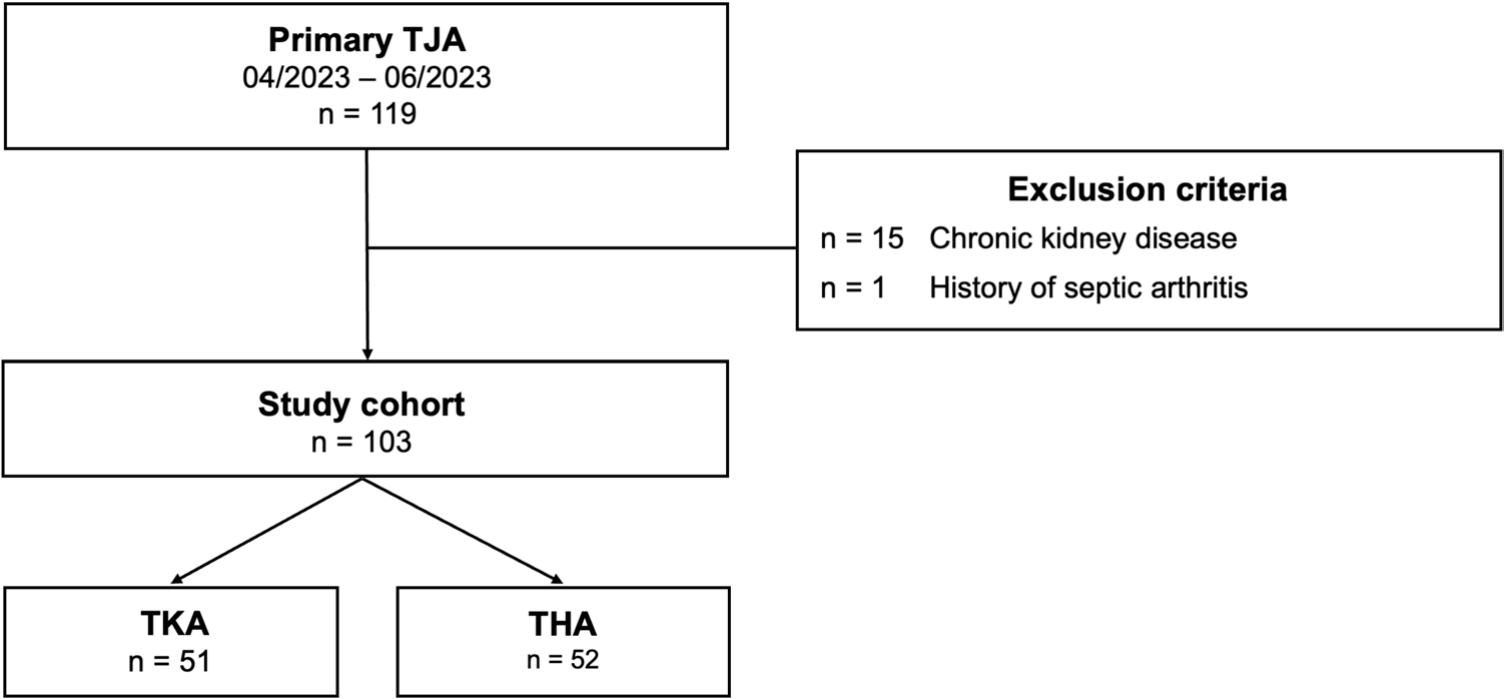
Flowchart of the study cohort. THA- total hip arthroplasty, TJA- total joint arthroplasty, TKA- total knee arthroplasty

Following institutional review board approval, this prospective, monocentric pilot study was conducted between April 2023 and June 2023. All patients undergoing primary THA or TKA were eligible. Exclusion criteria were defined as postinfectious arthritis, preexisting renal pathologies, or intravenous vancomycin therapy during inpatient hospital stay (Fig. 1).

103 consecutive patients were enrolled in the study, including 52 THA and 51 TKA procedures.

Demographic and clinical data were retrieved from the digital medical records, including age (at the time of the index procedure), sex, body mass index (BMI), ASA score, and relevant comorbidities.

The standard perioperative regimen included the application of a single shot antibiotic prophylaxis (2 g cefazolin) 30 min prior to skin incision. Standard prophylactic SSI protocols were followed including antiseptic skin preparation and application of an incision drape in both THA and TKA procedures.

One gram of vancomycin powder was applied intraarticular directly on the prosthesis. In TKA, a deep drain was placed prior to arthrotomy closure and was clamped for one hour following wound closure. Systemic serum vancomycin levels were analysed 24 and 48 h postoperatively using the VANC3 Gen.3^®^ immunoassay with the cobas analyser system (Roche Diagnostics GmbH, Mannheim, Germany) with a limit of detection of 1.5 μg/ml and a limit of quantitation of 4.0 μg/ml.

Ethical approval was obtained by the local ethics committee (#2023–173) and was conducted in accordance with the principles of the Declaration of Helsinki.

Normal distribution of data was tested using Shapiro–Wilk test. Group differences were calculated using Student’s t-test for normally distributed data and Mann–Whitney U-test for nonparametric data as appropriate. Paired data were analysed using Wilcoxon signed rank test. The Chi-square test was used to compare categorical data.

The statistical significance level was set at p < 0.05. Statistical analysis was performed using IBM SPSS Statistics 29 (SPSS Inc. Chicago, IL, USA). Figures were created using GraphPad Prism 5 (GraphPad Inc., La Jolla, CA, USA).

## Results

Of 103 patients included in this study, 64 were female (62.1%). Mean age at the time of the index procedure was 68.5 years, and mean ASA score was 2. Baseline characteristics did not reveal significant group differences (Table 1).

**Table 1:** Baseline characteristics of the study collective

	THA		TKA		p-value
	n = 52		n = 51		
Age, mean (± SD)	70.06	(± 11.60)	66.94	(± 6.42)	0.144
Gender					
Female, %	35	(67.31)	29	(56.86)	0.314
Male, %	17	(32.69)	22	(43.14)	
BMI [kg/m^2^], mean (± SD)	29.26	(± 6.46)	31.67	(± 9.70)	0.061
ASA-score, mean (range)	2	(1—3)	2	(1—3)	0.465
Serum creatinine [mg/dl] on admission, mean (± SD)	0.86	(± 0.22)	0.85	(± 0.17)	0.713
GFR [ml/min/1,73m^2^] on admission, mean (± SD)	78.29	(± 17.31)	80.14	(± 17.85)	0.598
Number of patients with detectable systemic vancomycin levels at 24 h, %	12	(23.08)	0	(0.00)	< 0.001*
Number of patients with detectable systemic vancomycin levels at 48 h, %	4	(7.69)	0	(0.00)	0.043*
Serum vancomycin level (24 h) [μg/ml], mean (range)	1.25	(0.00—7.00)	0.00	(0.00—0.00)	< 0.001*
Serum vancomycin level (48 h) [μg/ml], mean (range)	0.34	(0.00—4.80)	0.00	(0.00—0.00)	0.022*

^*^ indicates statistical significance

**Fig. 2:**
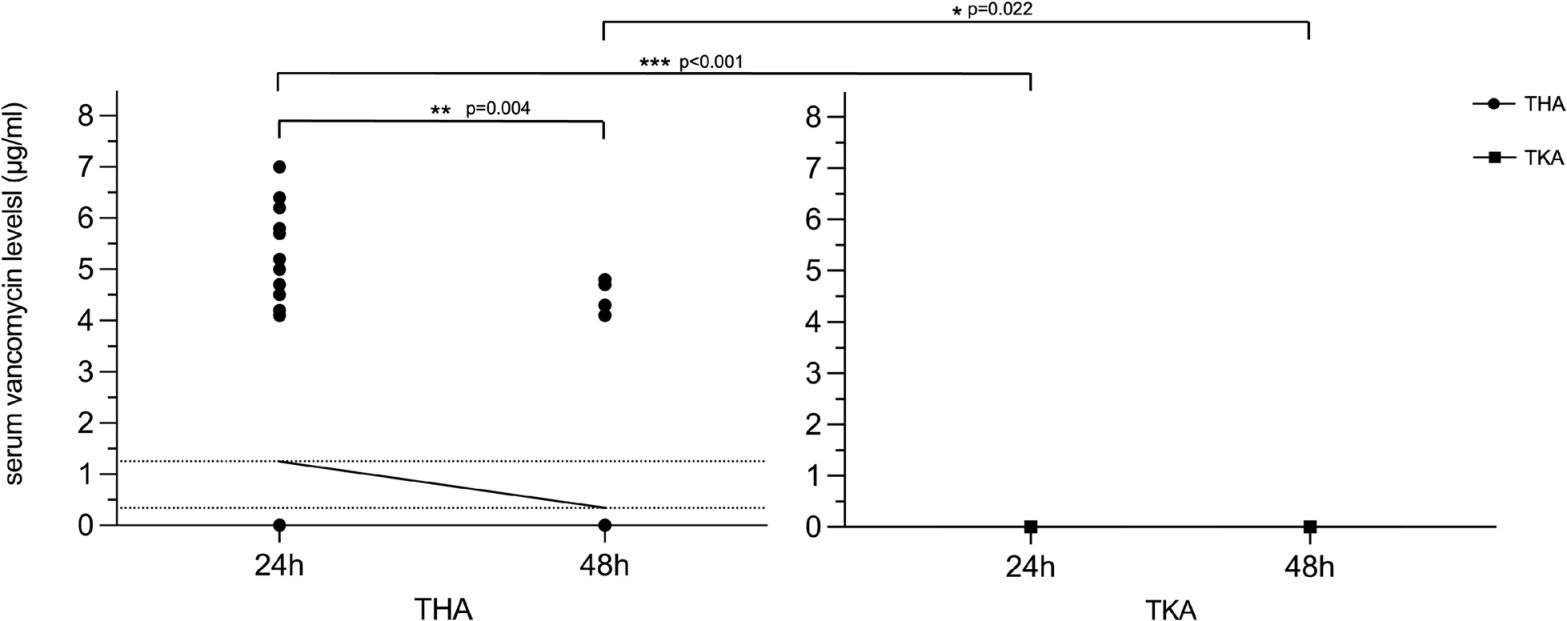
Serum vancomycin levels in THA and TKA patients. Statistical analysis was conducted using Student’s t-test and Wilcoxon ranked sum test for paired data. Dotted lines mark means at 24 h (1.25 μg/ml) and 48 h (0.34 μg/ml). Asterisks indicate statistical significance (*p < 0.05, **p < 0.010, ***p < 0.001)

Serum vancomycin levels were examined 24 h and 48 h postoperatively. In the whole study collective, systemic vancomycin levels could be detected in 12 (23.08%) patients after 24 h and 4 patients (7.69%) after 48 h. Mean serum vancomycin levels showed significant group differences at both time points (24 h: p < 0.001; 48 h: p = 0.044) with higher serum vancomycin concentrations in the THA cohort (Fig. 2).

In the THA subgroup, systemic vancomycin levels differed significantly between both time points (p = 0.004). The mean vancomycin levels in THA patients were 1.25 μg/ml (range 0.00—7.00 μg/ml) after 24 h and 0.34 μg/ml (range 0.00—4.80 μg/ml) after 48 h. Systemic vancomycin levels did not reach therapeutic levels in any patient. In the TKA cohort, no systemic vancomycin levels were detected in any case.

The mean follow-up showed a statistically significant difference between both groups (THA 5.9 months vs. TKA 5.4 months, p = 0.033). The overall PJI rate was 0.0% at a mean follow up of 5–6 months following the index procedure. The overall revision rate was 2.9% including one case presenting with superficial wound infection in the TKA collective, one periprosthetic fracture (Vancouver AG) in the THA collective and one revision due to early stem subsidence (Table 2).

**Table 2:** Postoperative complications

	THA		TKA		p-value
	n = 52		n = 51		
Postoperative follow up (months), mean (range)	5.87	(4—7)	5.43	(4—7)	0.033*
**Surgery-related complications**					
Postoperative complications					
Wound dehiscence, %	0	(0.00)	0	(0.00)	–
Superficial wound infection, %	0	(0.00)	1	(1.96)	0.495
Periprosthetic joint infection, %	0	(0.00)	0	(0.00)	–
Subsidence, %	1	(1.92)	0	(0.00)	0.505
Periprosthetic fracture, %	1	(1.92)	0	(0.00)	0.505
Revision surgery, %	2	(3.85)	1	(1.96)	0.507
**Systemic complications**					
Postoperative serum creatinine at 24 h, mean (± SD)	0.91	(0.30)	0.87	(0.20)	0.434
Postoperative serum creatinine at 48 h, mean (± SD)	0.88	(0.46)	0.84	(0.19)	0.648
Acute kidney injury (AKI) during inpatient stay, %	0	(0.00)	0	(0.00)	–

^*^ indicates statistical significance

Postoperative serum creatinine levels were equivalent between the two groups (p = 0.434, p = 0.648); no case of acute kidney injury (AKI) was observed in either group. No drug-related adverse events were detected in the whole study collective.

## Discussion

The most important finding of this present study is that no systemic vancomycin levels within the therapeutic range could be detected following intra-articular application of vancomycin powder in primary TJA.

Local VP application is a simple and cost-effective strategy sought to reduce the risk of PJI in THA and TKA procedures. By direct delivery of antibiotics to the targeted area, high concentrations in the surrounding tissues can be achieved with minimal systemic exposure and a reduced likelihood of systemic adverse effects [15].

Prophylactic topical vancomycin application has extensively been investigated in spine surgery [12, 20–22]. The results of these studies show a significant decrease in postoperative infection rates, thus local vancomycin application is considered an effective strategy for preventing surgical site infections.

Recently, topical VP has increasingly being used in primary total hip and total knee arthroplasty as an additional strategy for PJI prevention. Previous studies report different application strategies, including intrawound, intraarticular, and intraosseous administration of vancomycin in TJA [18, 23–26].

Harper et al. investigated the intraosseous (IO) application of vancomycin in THA surgery in a recent RCT, showing the feasibility of low-dose IO vancomycin to achieve equal and even superior tissue concentrations during surgery compared to intravenous (IV) application [24]. These results are in line with previously reported data by Young et al. published in 2017 [26]. The authors conclude that local VP application is a safe and effective strategy as it allows for an optimised timing of vancomycin administration during surgery in order to achieve the maximum local concentrations compared to IV application. In addition, the risk of systemic side effects is reduced due to local administration and lower vancomycin doses.

A more common approach is the intraarticular application of vancomycin powder before wound closure, which was also analysed in this present study [18, 23]. Although first evidence on the achieved local concentrations is promising, evidence on systemic vancomycin levels following intraarticular application is limited.

In this present study, 77% of the patients had undetectable systemic vancomycin levels, which is comparable to results published in the field of spine surgery [22]. Furthermore, no serum vancomycin levels within the therapeutic range of 15–20 μg/mL were detected in any case. As trough levels of more than 15–20 μg/mL may increase the risk of systemic side effects including nephrotoxicity, ototoxicity and anaphylaxis, our results suggest that intraarticular application of vancomycin powder leads to safe serum levels in TJA with a mean maximum level of 0.63 μg/mL 24 h postoperatively. The results of this present study are in line with evidence from Johnson et al., although these authors reported higher serum levels at 24 h (3.5 ± 3.5 μg/ml in THA and 3.5 ± 3.6 μg/ml in TKA) [23]. This is most likely attributable to different study protocols, as in their study, one gram of vancomycin was applied intraarticular and one gram was additionally applied to the superficial tissues before wound closure. Of note, recent systematic reviews and meta-analyses highlight the discrepancy of previously published study protocols, as vancomycin doses ranged from 0.5 to 3 g and localizations included intraarticular, subfascial and subcutaneous application of vancomycin powder [19, 27]. Moreover, in some studies, the protocol included prolonged IV antibiotics for 24 h, consequently the comparability of results is limited.

In our study, maximum serum vancomycin concentrations were observed 24 h postoperatively, and a decrease in vancomycin levels was monitored at the second analysis 48 h postoperatively. Johnson et al. repeatedly analysed the vancomycin levels at four timepoints (90 min, 3 h, 12 h, 24 h) and found relatively stable serum concentrations in the first 24 h postoperatively [23]. The authors assume that this is most likely attributable to the half-life of vancomycin of 6–12 h and a gradual absorption over an extended period of time. At the same time, intraarticular vancomycin levels exceeded the minimum inhibitory concentration and intrawound vancomycin levels were estimated to remain highly therapeutic for at least 24 up to 64 h.

Although these results are promising, a potential drawback that has to be kept in mind is that low systemic vancomycin levels potentially lead to the development of resistant bacterial strains, as international guidelines recommend systemic vancomycin levels > 10 μg/ml to avoid the development of resistances and 15–20 μg/ml for sufficient therapy [28].

Interestingly, serum vancomycin levels were significantly higher in the THA subgroup compared to TKA procedures. A possible reason may be that in TKA, VP was applied intracapsular, whereas in THA the joint capsule is not reconstructed, and the vancomycin powder is exposed to a larger area of vascularised skeletal muscle. In addition, in TKA procedures a deep drain was placed according to the internal intraoperative standard, which may lead to a partial loss of local antibiotics via the drain.

In this pilot study, the overall PJI incidence was 0.0% (0/103) at a mean follow up of 5.7 months postoperatively. Although this study is underpowered to detect differences in the risk of PJI, our preliminary findings are in line with evidence summarised by two recent large systemic reviews and meta-analyses showing a statistically significant reduction in PJI rates for the use of intraarticular vancomycin powder versus the control group for primary THA and TKA as well as revision procedures [19, 27]. In these studies, the overall infection rate pooled for primary and revision procedures was 0.87% vs. 1.65% and the overall infection rate for primary TJA was 0.57% vs. 1.39%, which was statistically significant.

Of note, some studies report a higher rate of sterile wound complications in patients receiving intrawound vancomycin powder compared to the control groups [17, 29].

Adequately powered, multicentre, prospective RCTs in greater patient cohorts are warranted to analyse PJI rates as well as safety related endpoints to determine the overall safety of topical vancomycin application.

This study has some limitations that are worth noting. The study is limited by the relatively small patient collective and limited follow up of 6 months. Furthermore, the time intervals of 24 h between the blood tests were relatively long, so no conclusions could be drawn about the exact course of the concentration curves.

## Conclusion

The results of our study suggest that intraarticular application of vancomycin powder in primary total hip and knee arthroplasty may be considered a safe adjunct for PJI prophylaxis, as we did not observe systemic vancomycin levels within the therapeutical range. Longitudinal studies in larger patient cohorts are warranted to further investigate the effect of intraarticular vancomycin application on PJI rates.

## Data Availability

The datasets generated and analyzed in this study are available from the corresponding author upon reasonable request.

## Abbreviations

ADR: Adverse drug reactions
AKI: Acute kidney injury
ASA: American society of anaesthesiologists score
BMI: Body mass index
IO: Intraosseous
IV: Intravenous
PJI: Periprosthetic joint infection
THA: Total hip arthroplasty
TJA: Total joint arthroplasty
TKA: Total knee arthroplasty
VP: Vancomycin powder
